# Social relationships and depression during the COVID-19 lockdown: longitudinal analysis of the COVID-19 Social Study

**DOI:** 10.1017/S0033291721000039

**Published:** 2021-01-13

**Authors:** Andrew Sommerlad, Louise Marston, Jonathan Huntley, Gill Livingston, Gemma Lewis, Andrew Steptoe, Daisy Fancourt

**Affiliations:** 1 Division of Psychiatry, University College London, London, UK; 2 Camden and Islington NHS Foundation Trust, London, UK; 3 Department of Primary Care and Population Health, University College London, London NW3 2PF, UK; 4 Department of Epidemiology and Public Health, University College London, London WC1E 6BT, UK; 5 Department of Behavioural Science and Health, University College London, London, UK

**Keywords:** Depression, social relationships, social support, social isolation, COVID-19, empathy

## Abstract

**Background:**

The coronavirus disease 2019 (COVID-19) pandemic led to measures that reduced social contact and support. We explored whether UK residents with more frequent or supportive social contact had fewer depressive symptoms during March−August 2020, and potential factors moderating the relationship.

**Methods:**

A convenience sample of UK dwelling participants aged ⩾18 in the internet-based longitudinal COVID-19 Social Study completed up to 22 weekly questionnaires about face-to-face and phone/video social contact frequency, perceived social support, and depressive symptoms using the PHQ-9. Mixed linear models examined associations between social contact and support, and depressive symptoms. We examined for interaction by empathic concern, perspective taking and pre-COVID social contact frequency.

**Results:**

In 71 117 people with mean age 49 years (standard deviation 15), those with high perceived social support scored 1.836 (1.801–1.871) points lower on PHQ-9 than those with low support. Daily face-to-face or phone/video contact was associated with lower depressive symptoms (0.258 (95% confidence interval 0.225–0.290) and 0.117 (0.080–0.154), respectively) compared to no contact. The negative association between social relationships and depressive symptoms was stronger for those with high empathic concern, perspective taking and usual sociability.

**Conclusions:**

We found during lockdown that those with higher quality or more face-to-face or phone/video contact had fewer depressive symptoms. Contact quality was more strongly associated than quantity. People who were usually more sociable or had higher empathy had more depressive symptoms during enforced reduced contact. The results have implications for COVID-19 and potential future pandemic management, and for understanding the relationship between social factors and mental health.

## Background

The novel coronavirus (2019-nCoV) related coronavirus disease 2019 (COVID-19) (Zhu et al., 2020) spread globally during early 2020, leading the World Health Organization to declare an international public health emergency on 30th January 2020 and a pandemic on 11th March 2020. Its highly infectious nature and the effect on individuals and health services caused many countries to initiate physical and social distancing measures and close non-essential businesses and services. On 16th March 2020, people in the UK were advised against all unnecessary social contact which included avoiding pubs, clubs, cinemas and restaurants (The Health Foundation, 2020). One week later on 23rd March 2020 a ‘lockdown’ was announced in the UK (UK Government Cabinet Office, 2020). These regulations specified that people should not leave home except for once-daily exercise, medical needs, essential shopping or work. Gatherings of more than two people were prohibited. This legislation was changed on 1st June 2020 when people living alone were permitted to meet with one other person outside, again on 13th June when two single-adult households were allowed to pair with one another and have unlimited contact, and on 4th July two households could meet indoors and multiple households could meet outdoors.

The potential detrimental impact on mental health of changes in social relationships resulting from the lockdown, including reduced frequency of social contact and insufficient social support, has been acknowledged (Luykx, Vinkers, & Tijdink, 2020) and noted in previous pandemic quarantines (Brooks et al., 2020). Social relationships can be measured in several ways, and commonly is divided into structural social relationships (i.e. the number and type of people with whom a person interacts), and functional aspects (meaning the qualitative experience of those interactions, such as perception of social support or loneliness) (Valtorta, Kanaan, Gilbody, & Hanratty, 2016). Having better structural or functional social relationships is linked to improved mental health. A systematic review of observational studies found that perceived support from others was associated with lowered risk of depression and depressive symptoms (Gariepy, Honkaniemi, & Quesnel-Vallee, 2016). Another added that having more extensive social networks was associated with lower risk of depression (Santini, Koyanagi, Tyrovolas, Mason, & Haro, 2015). These associations are found throughout the life-course including in older people (Santini et al., 2015) and children and adolescents (Loades et al., 2020).

The experience of social relationships changed during the COVID-19 pandemic. People turned to remote methods of communication, with increased use of phone calls – for example average call duration increased from 3.7 to 5.4 min from January to April 2020 in the UK (Ofcom, 2020) – and higher use of mobile messaging and video-calling in the UK and throughout Europe (Sun et al., 2020). It is important to consider whether these approaches to maintain relationships were associated with better mental health, as this may guide policy to reduce the impact of future periods of lockdown during COVID-19 and other pandemics.

Further, whilst there is evidence that richer social relationships are beneficial for mental health, it is unclear whether the unique circumstances of the COVID-19 pandemic, where reduction in social contact was imposed on all UK citizens, impacted the effect of social isolation on mental health. Socially active people may have suffered more from having social contact involuntarily reduced than those who had infrequent social contact prior to the pandemic. Furthermore, as reduced social contact was imposed on all for the purpose of promoting public health, it may have meant that psychological ability to think of others and conceive of the ‘greater good’ affected the experience of the lockdown. Higher empathic concern for others is associated with worse mental health, while higher ability to take the perspective of others is associated with better mental health (Lee, Brennan, & Daly, 2001; Tully, Ames, Garcia, & Donohue, 2016), so empathy may have moderated the effect of impaired social relationships on mental health, potentially creating high-risk groups for psychiatric distress during periods of lockdown.

Therefore, we aimed to test, in a large prospective study initiated at the start of the UK lockdown, our hypotheses that poor structural social relationships measured by frequency of face-to-face and phone or video contact and functional social relationships (measured by perceived social support) would be associated with more depressive symptoms and higher risk of depression. We used data with repeated weekly measures of social relationships (exposure) and depressive symptoms (outcome) to give more robust estimates of the associations and capture changes in social relationships as lockdown regulations changed. Additionally, we explored whether these associations would be strengthened in those with previous high sociability and high levels of empathic concern, and ameliorated in those with higher perspective-taking ability.

## Methods

### Study design and participants

UCL Research Ethics Committee [12467/005] approved the study and all participants gave informed consent. We conducted a longitudinal analysis of data from the COVID-19 Social Study (Fancourt, Steptoe, & Bu, 2020) of UK-dwelling participants aged 18 years and older. The COVID-19 Social Study started on 21st March 2020 to consider social and mental effects of the COVID-19 pandemic in the UK at the beginning of the lockdown. The study was a convenience sample promoted through several routes: large databases of people who had consented to be contacted about health research; United Kingdom Research and Innovation mental health research networks; media coverage; and targeted recruitment to increase representativeness to people from low income, low educational, unemployed backgrounds, and vulnerable groups. Full details of the study protocol are available at www.covidsocialstudy.org.

Participants were invited by email to complete online questionnaires using the Redcap online survey tool (https://www.project-redcap.org/). They could enrol in the study at any time after 21st March 2020 and, following their first extensive baseline questionnaire, received a weekly email until 21st August with a link to a shorter follow-up questionnaire. Participants who did not complete the weekly questionnaire received two reminder emails. If they still did not respond to the survey then they were counted as lost to follow-up.

Eligibility criteria for participants in our study were (1) being aged ⩾18 years, (2) joining the COVID-19 study any time between study inception on 21st March 2020 and 21st August 2020, the last date of weekly data collection, and (3) residing in the UK at the time of baseline questionnaire completion.

### Measures

#### Social relationship variables

Data about social relationships were collected weekly. For structural relationships, we asked:

(1) The number of days during the past week on which participants had at least 15 min of face-to-face social contact (including with those with whom they live).
(2) The number of days during the past week on which participants had at least 15 min of telephone or video social contact.

We used 15 min as the threshold for social contact as the prevalent advice regarding ‘social distancing’ at the time of study inception was to have fewer than 15 min of social contact with others, so longer social contact was likely to reflect meaningful social contact (Public Health England, 2020). We treated face-to-face and virtual contact separately, as responses to the two questions did not correlate (*r* = 0.05). Responses were 0–7, and we considered this as a continuous scale, and also categorised into none (0 days), some (1–6 days) and daily (7 days).

For functional relationships we used an adapted version of the six-item short form of Perceived Social Support Questionnaire, which was administered weekly. It has good psychometric properties (Kliem et al., 2015) including internal consistency (Cronbach's *α* 0.94) and we made minor adaptations to make the language more relevant to experiences during COVID-10 pandemic (online Supplementary appendix 1). Participants were asked to rate their agreement on a 5-point Likert scale from ‘not at all true’ to ‘very true’ with six statements about their feelings during the past week. Higher scores indicated more perceived social support. We considered this as a continuous variable and categorised scores into tertiles based on the distribution of the group (low <3, medium = 3 to 3.99, high ⩾4.)

#### Depressive symptoms

We measured depressive symptoms weekly using the Patient Health Questionnaire (PHQ-9) (Kroenke, Spitzer, & Williams, 2001), a standard instrument for assessing the severity of depressive symptoms in primary care settings with good psychometric properties including Cronbach's *α* 0.86–0.89. The questionnaire involves nine items, with responses on a Likert scale of 0−3 ranging from ‘not at all’ to ‘nearly every day’ with higher scores indicating more depressive symptoms. We used the scale as continuous, and also generated a binary variable as depressed/not depressed using a cut-off point of ⩾10 (Levis, Benedetti, & Thombs, 2019).

#### Potential moderators

*Empathic concern and perspective taking:* We used 14 questions from the Interpersonal Reactivity Index (IRI) (Davis, 1980) to evaluate self-reported empathic concern and perspective taking in one of the weekly surveys during week 13 of the study (13th–20th June 2020). Empathic concern and perspective taking have low−moderate correlation (Davis, 1980) and differential associations with depressive symptoms (Tully et al., 2016) so were treated separately in our analyses. The IRI is validated in the general population with internal consistency *α* 0.70–0.78, and is a measure of ‘trait-based’ empathy aiming to assess long-term, rather than situational, empathic responses, so does not focus on feelings related to the COVID-19 pandemic (Konrath, 2013). Participants were asked to rate their agreement with statements on a 5-point Likert scale ranging from ‘Does not describe me well’ to ‘Describes me very well’. Scores for the two subscales were averaged across each domain giving mean scores for empathic concern and perspective taking. We used this mean score and categorised tertiles based on the distribution in the sample (low <3.7, medium 3.7−4.19, high ⩾4.2 for empathic concern, and low <3.3, medium 3.3−3.99, high ⩾4 for perspective taking).

*Usual social contact:* We measured social contact prior to the COVID-19 pandemic at baseline with the question ‘Usually in your life, how often do you meet up with people face to face socially, not for work (e.g. friends, family, relatives or social events with colleagues)?’ Participants chose from response options ‘less than once weekly’, ‘once or twice per week’, or ‘three or more per week’.

#### Confounders

We used other variables from the baseline questionnaire which we considered from previous evidence to be potential confounders: age; gender (male, female, other/prefer not to say); ethnicity (White, other); highest educational attainment (lower secondary (GCSE/O-level or lower), higher secondary (A-level or equivalent), graduate or higher); living alone or living with others; marital status (cohabiting with partner or spouse, partner or spouse but living apart, divorced or widowed, single and never married); in employment/study or retired/not working; annual household income less or more than £ 30 000.

#### Analysis

We summarised the demographic characteristics of our sample, the social relationship variables, depression scale, empathic concern, perspective taking and usual pre-COVID social contact. We assessed the correlation between the social relationship variables using Spearman's rank test. We examined the amount of missing data and, as less than 0.5% throughout, we used complete case analysis.

*Association of social relationships with depressive symptoms and depression:* We examined the association between social relationship exposure variables and PHQ-9 scores using these data at all study time points between 21st March and 21st August 2020. We used mixed linear models with a random effect for intercept because data were clustered by individuals within study week, meaning that the coefficients derived from our analysis reflect the estimated mean difference in PHQ-9 score according to social relationship at that study time point. This method is preferable to a statistical approach taking just a single cross-sectional time point for each individual as they allowed us to use all available exposure (social relationships) and outcome (PHQ-9) weekly data within the period of data collection. This analytic method was preferable to modelling the trajectory of depressive symptoms based on social contact during the most restrictive part of the lockdown. We could instead continue to assess the association of social relationships and depression as lockdown restrictions changed, such as with the easing of regulations from June 2020 onwards.

We constructed models examining the association, in separate models, of face to face contact, phone/video contact, or social support with depressive symptoms, which were initially unadjusted (model 1), adjusted for age and sex (model 2), and additionally adjusted for education, employment status and income (model 3). A final model (model 4) additionally adjusted for amount of social contact (time varying), meaning that the model examining face-to-face contact additionally adjusted for phone/video contact, the model with phone/video contact additionally adjusted for face-to-face contact, and the model for social support adjusted for both face-to-face and phone/video contact. We examined the social relationship variables as both continuous and categorical. We repeated these analyses in mixed effect logistic regression models with depression as a binary outcome with a cut-point from PHQ-9 of 10, adjusted as in the linear regression models.

*Moderation by empathy and usual social contact:* We repeated model 3 of the analysis of social relationships (treated as continuous variables) and depressive symptoms. We added the potential moderating variables of empathic concern, perspective taking (as continuous variables) or usual social contact (as a categorical variable) and interaction terms of these with the social relationship variables (continuous) in separate models. We presented results for the association between the social relationship variables and depressive symptoms stratified according to tertiles of empathic concern, perspective taking, and the three categories of usual social contact.

## Results

We included 71 117 participants who answered 679 615 weekly questionnaires between 21st March and 21st August 2020; Table 1 details their demographic characteristics. Three quarters (53 026) were women and the mean age was 49 years [standard deviation (s.d.) 15]. In total, 66 673 (93.8%) were from White ethnic groups and 47 652 (67.0%) had attained graduate or higher educational level. Overall, 44 863 (63.1%) were married or cohabiting with their partner or spouse and 12 735 (17.9%) were living alone. In all, 46 333 (65.2%) were in employment or full-time education with the remainder not employed or retired.

**Table 1.** Characteristics of the sample (*N* = 71, 117)

Characteristic	Category	*n*/*N* (%) or Mean (s.d.)*
Age (years)	Mean (s.d.)	49.1 (14.9)*
	18–24	2872 (4.0)
	25–34	10858 (15.3)
	35–44	14 521 (20.4)
	45–54	15 602 (21.9)
	55–64	14 426 (20.3)
	65–74	10 230 (14.4)
	⩾75	2608 (3.7)
Gender	Female	53 026 (74.6)
	Male	17 756 (25.0)
	Other / prefer not to say	335 (0.5)
Ethnicity	White	66 673 (93.8)
	Other	4179 (5.9)
	*Missing*	265 (0.4)
Educational level	Lower secondary	10 576 (14.9)
	Higher secondary	12 889 (18.1)
	Graduate	47 652 (67.0)
Living situation	Lives alone	12 735 (17.9)
	Lives with others	58 382 (82.1)
Marital status	Cohabiting with partner/spouse	44 863 (63.1)
	Living apart from partner/spouse	4903 (6.9)
	Divorced/widowed	8973 (12.6)
	Single, never married	12 378 (17.4)
Employment	In employment	46 333 (65.2)
	Retired/not working	24 784 (34.8)
Household income	<£ 30 000	25 141 (35.4)
	⩾£ 30 000	39 422 (55.4)
	*Prefer not to say*	6554 (9.2)
Usual social contact	Less than once weekly	21 171 (29.8)
	Once or twice per week	24 239 (34.1)
	Three or more per week	25 428 (35.8)
	*Missing*	279 (0.4)
Mean interpersonal reactivity index scores	Empathic concern (*n* = 29 437)	4.0 (0.7)*
	Perspective taking (*n* = 29 573)	3.7 (0.7)*

s.d., standard deviation. Most of the values in the third column are n (%) but the ones with an asterisk, such as 'age' are mean (SD).

Of the 69 975 participants (Table 2) who gave baseline information about face-to-face contact, the median number of days with face-to-face contact at baseline was 7 [interquartile range (IQR) 3–7], with 44 676 (63.9%) having daily contact and 10 476 (15.0%) having no face-to-face contact during the preceding week. The median number of days on which participants (*n* = 70 074) had 15 min or more of phone/video contact was 4 (IQR 2–7) with 5964 (8.5%) reporting no days, 43 022 (61.4%) 1–6 days and 21 088 (30.1%) reporting daily phone/video contact. Median social support score was 4.0 (IQR 3.2–4.5) in the 68 784 who completed this questionnaire at baseline. The correlation between face-to-face and phone/video contact was 0.05; between face-to-face and social support was 0.27; and between phone/video contact and social support was 0.21. Mean PHQ-9 score was 7.1 (s.d. 6.1) and 20 235 of 69 671 participants (29.0%) scored 10 or more.

**Table 2.** Baseline structural and functional social relationships and depression

Baseline social relationships	Number of days during past week on which participant had face-to-face contact with another person for 15 min or more (including someone you live with)?	Score	Median (IQR)	7 (3, 7)
		None (0 days per week)	*n*/*N* (%)	10 476/69 875 (15.0)
		Some (1–6 days per week)	*n*/*N* (%)	14 723/69 875 (21.1)
		Daily	*n*/*N* (%)	44 676/69 875 (63.9)
	Number of days during past week on which participant had a phone or video call with another person for 15 min or more?	Score	Median (IQR)	4 (2, 7)
		None (0 days per week)	*n*/*N* (%)	5964/70 074 (8.5)
		Some (1–6 days per week)	*n*/*N* (%)	43 022/70 074 (61.4)
		Daily	*n*/*N* (%)	21 088/70 074 (30.1)
	Perceived social support	Score	Median (IQR)	4.0 (3.2, 4.5)
		Low	*n*/*N* (%)	14 057/68 784 (20.4)
		Medium	*n*/*N* (%)	20 132/68 784 (29.3)
		High	*n*/*N* (%)	34 595/68 784 (50.3)
Baseline depression	PHQ-9 score	Score	Mean (s.d.)	7.1 (6.1)
	Depression caseness (PHQ-9 score ⩾10)	Depressed	*n*/*N* (%)	20 235/69 671 (29.0)
		Non-depressed	*n*/*N* (%)	49 436/69 671 (71.0)

IQR, interquartile range; PHQ-9, Patient Health Questionnaire 9 item version; s.d. = standard deviation.

### Association of social relationships with depressive symptoms and depression

The results from our analyses of the association between social relationships and depressive symptoms are summarised in Table 3. Each additional day of face-to-face contact was associated with 0.052 [95% confidence interval (CI) 0.048–0.057] lower depression score adjusted for socio-demographic characteristics, and 0.051 (95% CI 0.047–0.056) when additionally adjusted for phone/video contact. Having daily contact was associated with 0.258 (95% CI 0.225–0.290) lower PHQ-9 score than having no face-to-face contact, [0.247 (0.215–0.280) when adjusted for phone/video contact].

**Table 3.** Association of structural and functional social relationships with depressive symptoms

		Model 1, unadjusted		Model 2, Adjusted for age and sex		Model 3, Model 2 + adjusted for education, employment status and income		Model 4, Model 3 + adjusted for face-to-face and/or phone/video contact	
		Coefficient	(95% CI)	Coefficient	(95% CI)	Coefficient	(95% CI)	Coefficient	(95% CI)
Number of days during past week on which participant had face-to-face contact with another person for 15 min or more	Per 1 day more contact	**−0.051**	**(−0.056 to −0.047)**	**−0.059**	**(−0.064 to −0.055)**	**−0.052**	**(−0.057 to −0.048)**	**−0.051**	**(−0.056 to −0.047)**
	None (0 days per week)	Reference		Reference		Reference		Reference	
	Some (1–6 days per week)	−0.003	(−0.031 **to** 0.026)	−0.021	(−0.049 **to** 0.008)	−0.012	(−0.042 **to** 0.018)	−0.010	(−0.040 **to** 0.020)
	Daily	**−0.251**	**(−0.282 to −0.220)**	**−0.302**	**(−0.333 to −0.271)**	**−0.258**	**(−0.290 to −0.225)**	**−0.247**	**(−0.280 to −0.215)**
Number of days during past week on which participant had phone or video call contact with another person for 15 min or more	Per 1 day more contact	**−0.030**	**(−0.034 to −0.025)**	**−0.033**	**(−0.038 to −0.029)**	**−0.026**	**(−0.031 to −0.021)**	**−0.023**	**(−0.028 to −0.018)**
	None (0 days per week)	Reference		Reference		Reference		Reference	
	Some (1–6 days per week)	**−0.078**	**(−0.106 to −0.049)**	**−0.094**	**(−0.122 to −0.066)**	**−0.061**	**(−0.090 to −0.031)**	**−0.043**	**(−0.073 to −0.013)**
	Daily	**−0.162**	**(−0.196 to −0.127)**	**−0.186**	**(−0.220 to −0.151)**	**−0.145**	**(−0.182 to −0.108)**	**−0.117**	**(−0.154 to −0.080)**
Perceived social support	Per one point increase	**−1.020**	**(−1.034 to −1.005)**	**−1.057**	**(−1.071 to −1.043)**	**−1.033**	**(−1.048 to −1.018)**	**−1.035**	**(−1.050 to −1.020)**
	Low	Reference		Reference		Reference		Reference	
	Medium	**−0.939**	**(−0.967 to −0.912)**	**−0.983**	**(−1.010 to −0.955)**	**−0.962**	**(−0.991 to −0.933)**	**−0.956**	**(−0.986 to −0.927)**
	High	**−1.819**	**(−1.851 to −1.786)**	**−1.890**	**(−1.922 to −1.857)**	**−1.848**	**(−1.882 to −1.814)**	**−1.836**	**(−1.871 to −1.801)**

***Notes*:** Coefficient represents mean number of points on PHQ-9 higher or lower according to social relationship characteristic. Bold results indicate *p* < 0.05.

Each additional day of phone/video contact was associated with 0.026 (95% CI 0.021–0.031) lower PHQ-9 score [0.023 (95% CI 0.018–0.028) when adjusted for amount of face-to-face contact]. Having some or daily phone or video contact was associated with lower depressive symptoms compared to no phone or video contact when adjusted for face-to-face contact; PHQ-9 scores 0.043 (95% CI 0.013–0.073), and 0.117 (95% CI 0.080–0.154) lower for some and daily contact, respectively, compared to no phone/video contact.

Higher reported perceived social support was associated with lower depressive symptoms: for each additional point, PHQ-9 score was 1.033 (95% CI 1.018–1.048) lower in models adjusting for sociodemographic characteristics and 1.035 (95% CI 1.020–1.050) when additionally adjusting for amount of face-to-face and phone/video contact. Those in the highest social support tertile scored a mean 1.836 (95% CI 1.801–1.871) points lower on PHQ-9 than those in the lowest tertile in fully adjusted models.

A similar pattern of results was seen in multilevel logistic regression models with a binary depression outcome (Table 4). The odds ratio for depression for people having daily compared to no face-to-face social contact was 0.712 (95% CI 0.747–0.678) in models adjusting for sociodemographic characteristics and phone/video contact. Those with daily phone/video contact compared to no contact had odds ratio for depression 0.825 (95% CI 0.779–0.873) in fully adjusted models. Reporting high, compared to low, social support was associated with 0.145 (95% CI 0.138–0.152) odds ratio for depression, adjusted for sociodemographic status and face-to-face and phone/video contact.

**Table 4.** Association of structural and functional social relationships with depression (odds ratio for depression)

		Model 1, unadjusted		Model 2, Adjusted for age and sex		Model 3, Model 2 + adjusted for education, employment status and income		Model 4, Model 3 + adjusted for face-to-face and/or phone/video contact	
		Odds ratio	(95% CI)	OR	95% CI	OR	95% CI	OR	95% CI
Number of days during past week on which participant had face-to-face contact with another person for 15 min or more	Per 1 day more contact	**0.937**	**(0.931–0.943)**	**0.921**	**(0.915–0.927)**	**0.937**	**(0.931–0.944)**	**0.939**	**(0.933–0.945)**
	None (0 days per week)	Reference		Reference		Reference		Reference	
	Some (1–6 days per week)	**0.954**	**(0.913–0.997)**	**0.912**	**(0.873–0.954)**	**0.945**	**(0.903–0.990)**	**0.945**	**(0.903–0.990)**
	Daily	**0.701**	**(0.669–0.734)**	**0.625**	**(0.596–0.655)**	**0.703**	**(0.669–0.738)**	**0.712**	**(0.678–0.747)**
Number of days during past week on which participant had phone or video call contact with another person for 15 min or more	Per 1 day more contact	**0.949**	**(0.942–0.955)**	**0.942**	**(0.935–0.948)**	**0.956**	**(0.949–0.963)**	**0.959**	**(0.952–0.966)**
	None (0 days per week)	Reference		Reference		Reference		Reference	
	Some (1–6 days per week)	**0.861**	**(0.823–0.900)**	**0.830**	**(0.794–0.868)**	**0.890**	**(0.849–0.932)**	**0.906**	**(0.864–0.949)**
	Daily	**0.777**	**(0.736–0.819)**	**0.737**	**(0.700–0.778)**	**0.799**	**(0.755–0.845)**	**0.825**	**(0.779–0.873)**
Perceived social support	Per one point increase	**0.349**	**(0.342–0.355)**	**0.332**	**(0.326–0.338)**	**0.356**	**(0.349–0.363)**	**0.357**	**(0.350–0.364)**
	Low	Reference		Reference		Reference		Reference	
	Medium	**0.387**	**(0.372–0.402)**	**0.362**	**(0.349–0.376)**	**0.387**	**(0.372–0.403)**	**0.393**	**(0.377–0.409)**
	High	**0.137**	**(0.131–0.144)**	**0.122**	**(0.117–0.128)**	**0.141**	**(0.134–0.147)**	**0.145**	**(0.138–0.152)**

Bold results indicate *p* < 0.05.

### Moderation by empathy and usual social contact

**Fig. 1.**
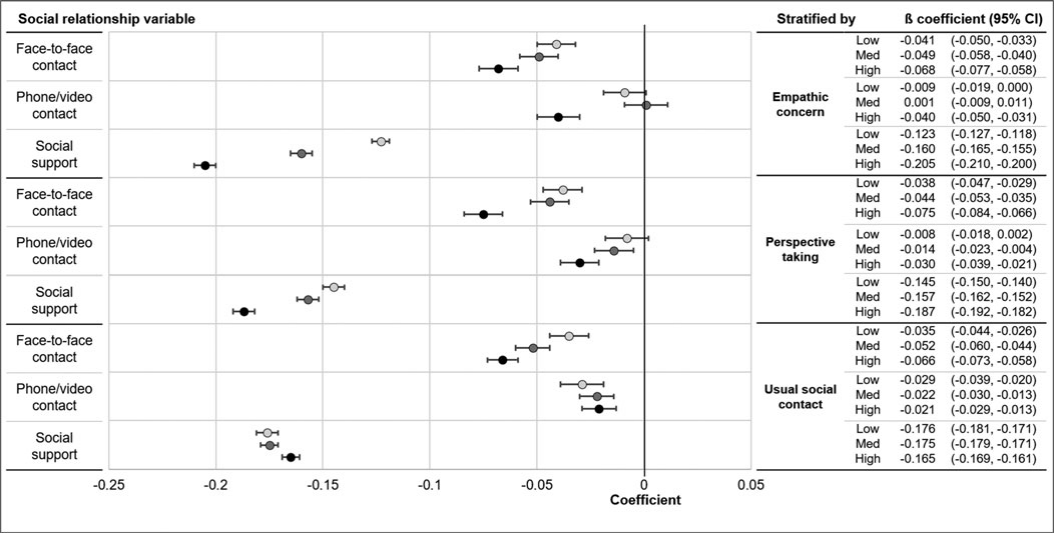
Association of face-to-face contact, phone/video contact and social support with depressive symptoms, stratified by empathic concern, perspective taking and usual social contact. *Notes*: Coefficients indicate the number of points difference on PHQ-9 depression scale associated with one point higher social relationship score, stratified by level of empathic concern, perspective taking and social contact. All analyses are adjusted for age, sex, education, employment status and income.

We repeated analyses with interaction terms for potential moderators, results are described in Fig. 1, which shows the number of points difference on PHQ-9 depression scale associated with one point higher social relationship score, stratified by level of empathic concern, perspective taking and social contact. We found an interaction for both empathic concern and perspective taking in the negative relationship between face-to-face contact and depressive symptoms, with a higher association in participants with higher empathy. In the 29 567 people who completed the empathic concern section of the IRI, those with high empathic concern scored 0.068 (95% CI 0.058–0.077) points lower on PHQ-9 for each additional day of social contact, compared to those with low empathic concern who scored 0.041 (95% CI 0.033–0.050) lower with more social contact. In the 29 701 people who completed the perspective taking questionnaire, participants with high perspective taking scores scored 0.075 (95% CI 0.066–0.084) points lower on depressive symptoms for each additional day of face-to-face contact, whereas those with low perspective taking scored 0.038 (95% CI 0.029–0.047) points lower with more face-to-face contact. Higher empathic concern and perspective taking however was linked to smaller association between perceived social support and depressive symptoms. Those with higher empathic concern and perspective taking reported lower depressive symptoms with additional social support, compared to those with lower scores on the empathy variables.

We found evidence of interaction between retrospectively self-reported pre-COVID social contact and the relationship between face-to-face contact and depressive symptoms. Those who usually had three or more social meetings per week scored 0.066 (95% CI 0.058–0.073) points lower on the depression scale with each extra day of social contact during the lockdown, whereas those who usually met socially with people less than once weekly experienced less effect on depression with social contact, scoring 0.035 (95% CI 0.026–0.044) points lower on the depression scale for each additional social contact. Small differences were seen in the associations between phone/video contact and depressive symptoms and social support and depressive symptoms according to level of usual pre-COVID social contact. In these analyses those who usually had more social contact experienced a marginally smaller effect of having more phone/video contact or social support on depressive symptoms than those who usually had infrequent social contact.

## Discussion

In this large longitudinal study during the first COVID-19-related lockdown in the UK, we found that those with higher levels of perceived social support had markedly lower depressive symptoms and depression risk. We also report that both more frequent face-to-face and phone/video contact were associated with lower depressive symptoms, including when mutually adjusted for one another, and in-person contact had more effect than digital contact. These relationships persisted when adjusted for the amount of actual weekly face-to-face or digital social contact.

Our main finding that having more or better social relationships was associated with lower depressive symptoms and risk of depression is consistent with the literature. A systematic review of 51 studies, of which 23 were prospective, reported strong consistent findings that having more perceived support from others and more extensive social networks was associated with lower risk of depression (Santini et al., 2015). Establishing the direction of this association can be challenging. Many studies suggest that having positive and enjoyable social experiences and recalling socially rewarding information is linked to lower depressive symptoms (Lewis et al., 2017), that experiencing loneliness is linked to higher threat vigilance which may promote depressive cognitions (Hawkley & Cacioppo, 2010), and that social support may moderate the effect of stressful life events on health (Cobb, 1976). This has been confirmed through studies tracking experiences longitudinally, including a recent study of UK older adults that found greater risk of depression up to 12 years later in those reporting loneliness (Lee et al., 2020). However, the association may be bidirectional as depressive symptoms such as reduced capacity for enjoyment, interest and concentration, and impaired self-esteem and self-confidence may impair social relationships or a person's perception of them. Therefore, associations between self-rated structural and functional aspects of relationships may partly be a manifestation of depressive symptomatology. What is unique in this study is that quantity of social relationship was affected by law for everyone in the UK. While people who are depressed may usually decide not to see people socially, during this period everyone had their social contact restricted.

Our findings also have specific relevance to the social context of the COVID-19 pandemic, echoing some previous studies. For example, one cross-sectional study of 7127 UK older adults aged 70 on average who self-reported whether their mental health had changed, found that loneliness was associated with reporting a worsening of depression and anxiety symptoms, although this study did not objectively identify change in mental health (Robb et al., 2020). In our study, the association was relatively modest; the minimum difference on the PHQ-9 considered to indicate a clinically meaningful improvement in response to treatment being between 2.8 and 5 points (Löwe, Unützer, Callahan, Perkins, & Kroenke, 2004). However, the magnitude of the association for depression risk was substantial – those with daily face-to-face contact had 29% lower risk of depression, and risk was 18% lower for video contact. Associations with depression were stronger for the functional rather than structural measure of social relationships and persisted when adjustment for structural relationships was included: those with medium and high perceived social support had 61 and 85% lower depression risk, respectively, than those with low social support. This may indicate that it is the quality, rather than quantity, of social relationships which matters most, or may reflect the overlap between depressive symptomatology and negative judgment about support from others. Importantly, our study also provided new findings on the relationship between face-to-face *v*. virtual communication and mental health.

Some studies conducted prior to the COVID-19 pandemic have suggested similar benefits of video contact. In a study of young adults, both face to face and video enjoyable interactions were associated with improvements in self-esteem but face-to-face interaction had a stronger effect (Subrahmanyam, Frison, & Michikyan, 2020). A further study of US college students reported that affect improved more following in-person communication than digital communication (Holtzman, DeClerck, Turcotte, Lisi, & Woodworth, 2017) and another study reported that relationship strength was higher following face-to-face communication compared to video, audio and text communication (Sherman, Michikyan, & Greenfield, 2013). Our study suggests that these experimental findings apply in a pandemic-enforced lockdown. Face-to-face contact appeared to confer greater benefit, but when in-person communication was taken into account, remote communication remained beneficial. The use of social media for communication is common, with 88% of young adults reporting using it in 2018 (Pew Research Center, 2018), and the use has increased during the COVID-19 pandemic (Ofcom, 2020). Social information processing theory suggests that frequent use of digital communication over time allows users to convey and process personal information effectively, despite fewer non-verbal cues than in face-to-face settings (Walther, 2011) so remote communication is likely to be beneficial. It may also be that our study's measure of face-to-face social contact during the lockdown captured contact with close family and friends, as contact was most likely with cohabiting people, whereas digital contact was more likely to be with more distant contacts. Therefore, the stronger association of in-person rather than digital communication may have partly reflected the relationship, rather than the method of contact.

A secondary aim of our study was to explore whether having more empathy for others or usual sociability affected these associations; the first known study to do this. We found that higher empathic concern for others was linked to a stronger effect of structural and functional social relationships on increasing depression, which is consistent with the associations between higher empathic concern and depressive symptoms (O'Connor, Berry, Lewis, Mulherin, & Crisostomo, 2007). Deprivation of social relationships may be felt more strongly for those who empathise more with others. Contrary to our hypothesis, higher perspective taking did not attenuate the detrimental effect of social isolation on mental health and instead, strengthened the relationship. Our hypothesis was based on studies reporting an inverse relationship between perspective taking and depressive symptoms, usually in caring groups such as family carers and healthcare workers (Hollinger-Samson & Pearson, 2000; Jütten, Mark, & Sitskoorn, 2019; Lee et al., 2001; Tully et al., 2016), suggesting that ability to conceptualise another's perspective may moderate the experience of their distress. However, our finding may indicate that people who had others' perspectives in mind found the lockdown more difficult as, in these circumstances, they had little agency to act to alleviate others' situations.

Finally, we report a novel finding that the effect of impaired structural and functional relationships during the lockdown was associated more strongly with depression in people who were previously more socially active. Several studies have suggested that unmarried people who live alone have wider and more active social networks and these have a greater impact on wellbeing for single people than for those in relationships (Cwikel, Gramotnev, & Lee, 2006; Ermer & Proulx, 2019; Stokes & Moorman, 2018). In our sample, those with less daily face-to-face contact were likely to be living alone and therefore unable to mix socially with the wide range of people with whom they would normally do so, and this may have been detrimental to mental health.

### Limitations

While our large sample size covered an extensive range of sociodemographic characteristics, it was not nationally representative with some groups being underrepresented, for example those from lower sociodemographic groups and minority ethnic groups. However, the potential bias in selection is less relevant for examining risk factor-outcome associations (Batty et al., 2014). All variables were by self-report, so negative perspectives common in depression may have influenced report of structural and functional relationships, which would likely overestimate the association.

The questionnaire design, whereby respondents had to answer all questions to proceed to the next page, meant that there was little missing data for the different questionnaire domains, but participation varied longitudinally, with around 10% answering only one questionnaire, and only 10% answering all the weekly questionnaires. Our analysis did not account for attrition which may have been higher in those with depressive symptoms, and we could only examine moderation by empathy in the smaller sample of participants who answered that weekly questionnaire. Participants also joined at different stages, with around 40% joining within the first week of the survey in late March 2020, and others joining at any subsequent point. Our analytic approach allowed us to make use of all repeated exposure and outcome variables, and this was particularly relevant for the circumstances of lockdown whereby social contact with others could vary markedly from week-to-week as new legislation came into force, but our approach makes it difficult to be certain of the direction of association and reverse causality may have affected our results. Finally, we lacked detail about the nature of social interactions, such as the duration and intensity of social contact and there is the potential for residual confounding from unmeasured confounders.

### Clinical and research implications

This large longitudinal study of structural and functional relationships and depressive symptoms throughout the first COVID-19-related lockdown in the UK supports the existing literature that both structural and functional aspects of social relationships are associated with better mental health. Our study adds that good quality and supportive face-to-face contact with others is likely to be most beneficial but that, even when this is not available or permitted, phone and video contact may be beneficial. We also find that the impact of social isolation may be most hard-felt for those who are usually socially active and more empathic.

These findings have immediate clinical and public health relevance. The UK has already had further periods of physical and social distancing due to COVID-19 and these are likely in the future, so identifying high-risk groups for negative effects is important. Social isolation is associated with other adverse cognitive (Sommerlad, Sabia, Singh-Manoux, Lewis, & Livingston, 2019) and physical effects (Holt-Lunstad, Smith, Baker, Harris, & Stephenson, 2015), so public health policy should facilitate social contact, where possible, to alleviate the burden on mental health especially for those who live alone and are accustomed to contact with others. Individuals should use digital methods of communication when in-person meetings are limited. There is need for actions to improve social connectedness throughout this and potential future pandemics (Smith, Steinman, & Casey, 2020) to reduce the potential for mental illness arising from social isolation.

## Supplementary material

For supplementary material accompanying this paper visit https://doi.org/10.1017/S0033291721000039. click here to view supplementary material

## Data sharing

Data are from the COVID-19 Social Study which is currently not publicly available, but interested collaborators may contact the study team.
