# Supplementary material for: Social relationships and depression during the COVID-19 lockdown: longitudinal analysis of the COVID-19 Social Study
Source: Psychol Med. 2021 Jan 13:1–10. doi: 10.1017/S0033291721000039 (PMC7844174; doi:10.1017/S0033291721000039)
Supplement: Supplementary file 1 [file S0033291721000039sup001.docx]

## Appendix 1 – Comparison of items in the original and revised Perceived Social Support Questionnaire

| Original | Adapted for COVID-19  In the past week, I feel… |
| --- | --- |
| I experience a lot of understanding and security from others | I have experienced a lot of understanding and support from others |
| I know a very close person whose help I can always count on | I have a very close person whose help I can always count on |
| If necessary, I can easily borrow something I might need from neighbours or friends | If necessary, I can easily borrow something I need from neighbours or friends |
| I know several people with whom I like to do things | I have people with whom I can spend time and do things together |
| When I am sick, I can without hesitation ask friends and family to take care of  important matters for me | If I get sick, I have friends and family who will take care of me |
| If I am down, I know to whom I can go without hesitation | If I am feeling down, I have people I can talk to without hesitation |

**Citation for Perceived social support questionnaire:**

Kliem S, Mößle T, Rehbein F, Hellmann DF, Zenger M, Brähler E. A brief form of the Perceived Social Support Questionnaire (F-SozU) was developed, validated, and standardized. *Journal of clinical epidemiology* 2015; **68**(5): 551-62
